# The genome sequence of the Antarctic lanternfish, *Electrona antarctica *(Günther, 1878)

**DOI:** 10.12688/wellcomeopenres.23803.2

**Published:** 2025-07-25

**Authors:** Iliana Bista, Martin Collins, Wellcome Sanger Institute Tree of Life Management, Samples and Laboratory team, Wellcome Sanger Institute Scientific Operations: Sequencing Operations, Wellcome Sanger Institute Tree of Life Core Informatics team, Tree of Life Core Informatics collective

**Affiliations:** 1 Senckenberg Research Institute and Natural History Museum, Frankfurt, Germany; 2 British Antarctic Survey, NERC, Cambridge, England, UK

**Keywords:** Electrona antarctica, Antarctic lanternfish, genome sequence, chromosomal, Myctophiformes

## Abstract

We present a genome assembly from an individual female *Electrona antarctica* (the Antarctic lanternfish; Chordata; Actinopterigii; Myctophiformes; Myctophidae). The genome sequence has a total length of 1,427.40 megabases. Most of the assembly is scaffolded into 24 chromosomal pseudomolecules. The mitochondrial genome has also been assembled and is 20.02 kilobases in length.

## Species taxonomy

Eukaryota; Opisthokonta; Metazoa; Eumetazoa; Bilateria; Deuterostomia; Chordata; Craniata; Vertebrata; Gnathostomata; Teleostomi; Euteleostomi; Actinopterygii; Actinopteri; Neopterygii; Teleostei; Osteoglossocephalai; Clupeocephala; Euteleosteomorpha; Neoteleostei; Eurypterygia; Ctenosquamata; Myctophata; Myctophiformes; Myctophidae; *Electrona*; *Electrona antarctica* (Günther, 1878) (NCBI:txid206093).

## Background

The Antarctic lanternfish *Electrona antarctica*, is a member of the Myctophidae family of fish (Order: Myctophiformes). Myctophidae are mesopelagic fish comprising over 250 species and constitute one of the most dominant fish groups throughout the global oceans ( Catul *et al.*, 2011; Collins *et al.*, 2012). Myctophids undergo extensive vertical migration moving between depths of 50 and 400 m during different times of the day ( Hulley & Duhamel, 2011). Feeding in myctophids is aimed mostly at mesozooplankton, including amphipods, copepods, and euphasids (krill), while they are themselves major feeding targets of penguins, other fish (e.g. toothfish), seals and other higher predators ( Collins *et al.*, 2012).

Populations of the Antarctic lanternfish *E. antarctica* occur in the Southern Ocean (SO), with a distribution including the Antarctic Polar front and extending all the way to the far South ( Hulley, 1990). *E. antarctica* is considered a key species, among the already dominant Myctophidae family, due to its high abundance and wide distribution. The conservation status of this species is currently listed as a “Least concern” in the IUCN red list ( Williams, 2019).

Here we present a genome assembly generated using an adult specimen collected from the Antarctic Polar Front area, during the JR19001 cruise onboard the James Clark Ross research vessel.

## Genome sequence report

**Figure 1.**
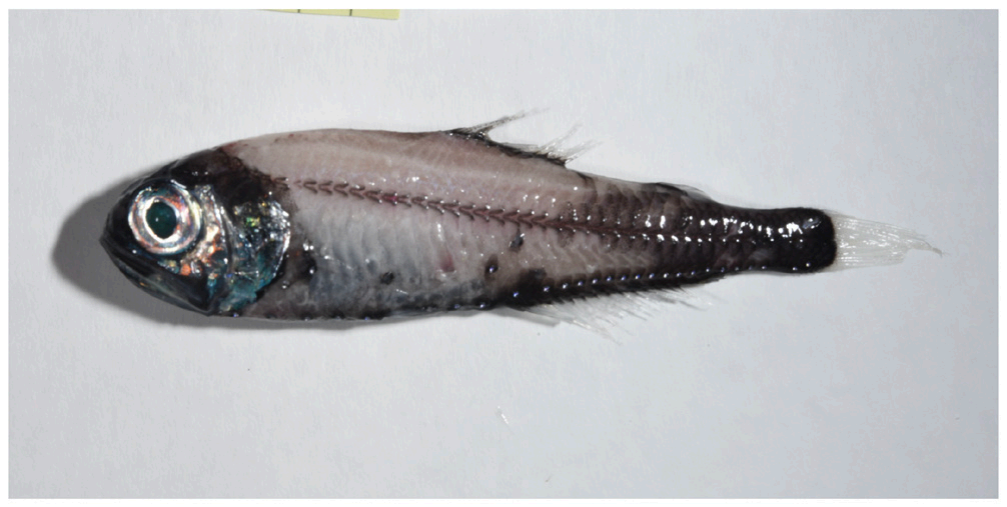
Photograph of the *Electrona antarctica* (fEleAnt2) specimen used for genome sequencing.

The genome of a specimen of *Electrona antarctica* ( Figure 1) was sequenced using Pacific Biosciences single-molecule HiFi long reads, generating a total of 54.73 Gb (gigabases) from 6.17 million reads, providing approximately 43-fold coverage. Primary assembly contigs were scaffolded with chromosome conformation Hi-C data, which produced 251.34 Gb from 1,664.50 million reads, yielding an approximate coverage of 176-fold. Specimen and sequencing details are provided in Table 1.

**Table 1.** Specimen and sequencing data for *Electrona antarctica*.

Project information			
**Study title**	*Electrona antarctica* (Antarctic lanternfish)		
**Umbrella BioProject**	PRJEB60649		
**Species**	*Electrona antarctica*		
**BioSample**	SAMEA8748807		
**NCBI taxonomy ID**	206093		
Specimen information			
**Technology**	**ToLID**	**BioSample accession**	
**PacBio long read sequencing**	fEleAnt2	SAMEA8748807	
**Hi-C sequencing**	fEleAnt3	SAMEA8748808	
**RNA sequencing**	fEleAnt3	SAMEA8748826	
Sequencing information			
**Platform**	**Run accession**	**Read count**	**Base count (Gb)**
**Hi-C Illumina NovaSeq 6000**	ERR11040177	1.66e+09	251.34
**PacBio Sequel IIe**	ERR11029670	2.18e+06	19.29
**PacBio Sequel IIe**	ERR11029669	9.39e+05	10.84
**PacBio Sequel IIe**	ERR11029671	1.47e+06	10.94
**PacBio Sequel IIe**	ERR11029672	1.58e+06	13.66
**RNA Illumina HiSeq 4000**	ERR11040178	4.33e+07	6.54
**RNA Illumina NovaSeq 6000**	ERR12245545	9.15e+07	13.82

**Figure 2.**
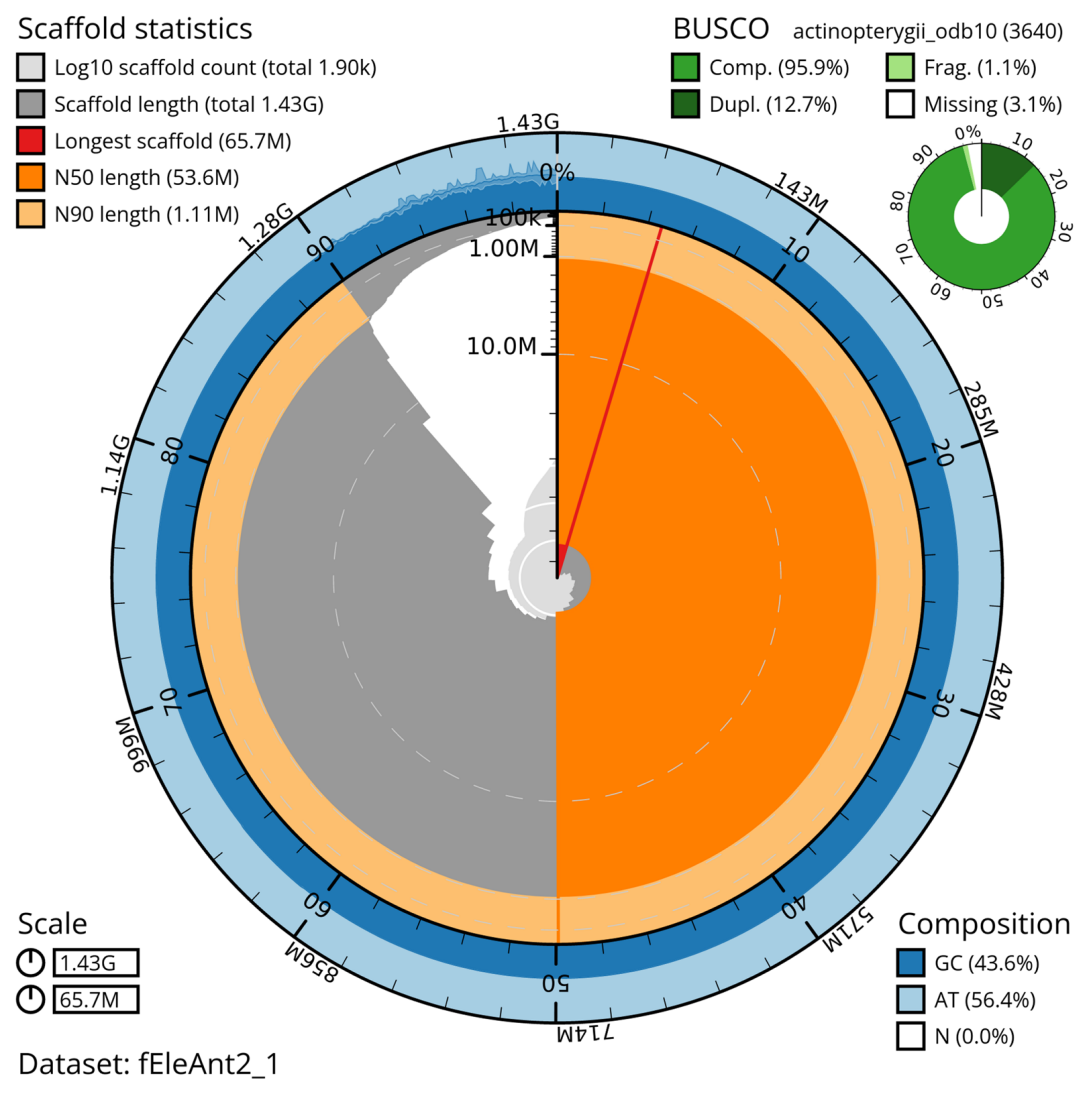
Genome assembly of *Electrona antarctica*, fEleAnt2.1: metrics. The BlobToolKit snail plot shows N50 metrics and BUSCO gene completeness. The main plot is divided into 1,000 size-ordered bins around the circumference with each bin representing 0.1% of the 1,427,445,741 bp assembly. The distribution of scaffold lengths is shown in dark grey with the plot radius scaled to the longest scaffold present in the assembly (65,725,131 bp, shown in red). Orange and pale-orange arcs show the N50 and N90 scaffold lengths (53,633,514 and 1,105,000 bp), respectively. The pale grey spiral shows the cumulative scaffold count on a log scale with white scale lines showing successive orders of magnitude. The blue and pale-blue area around the outside of the plot shows the distribution of GC, AT and N percentages in the same bins as the inner plot. A summary of complete, fragmented, duplicated and missing BUSCO genes in the actinopterygii_odb10 set is shown in the top right. An interactive version of this figure is available at https://blobtoolkit.genomehubs.org/view/fEleAnt2_1/dataset/fEleAnt2_1/snail.

**Figure 3.**
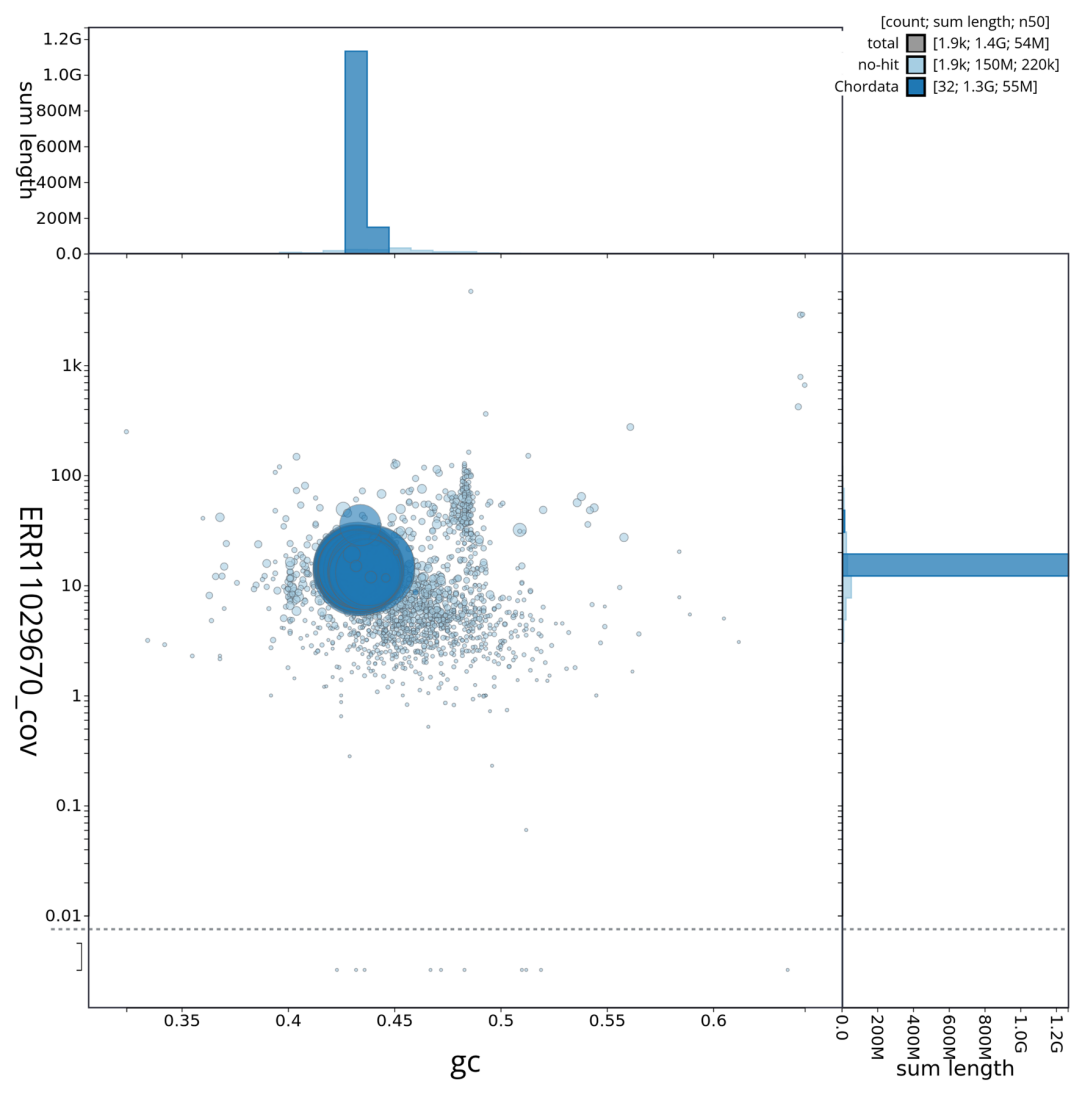
Genome assembly of *Electrona antarctica*, fEleAnt2.1: BlobToolKit GC-coverage plot. Sequences are coloured by phylum. Circles are sized in proportion to sequence length. Histograms show the distribution of sequence length sum along each axis. An interactive version of this figure is available at https://blobtoolkit.genomehubs.org/view/fEleAnt2_1/dataset/fEleAnt2_1/blob.

**Figure 4.**
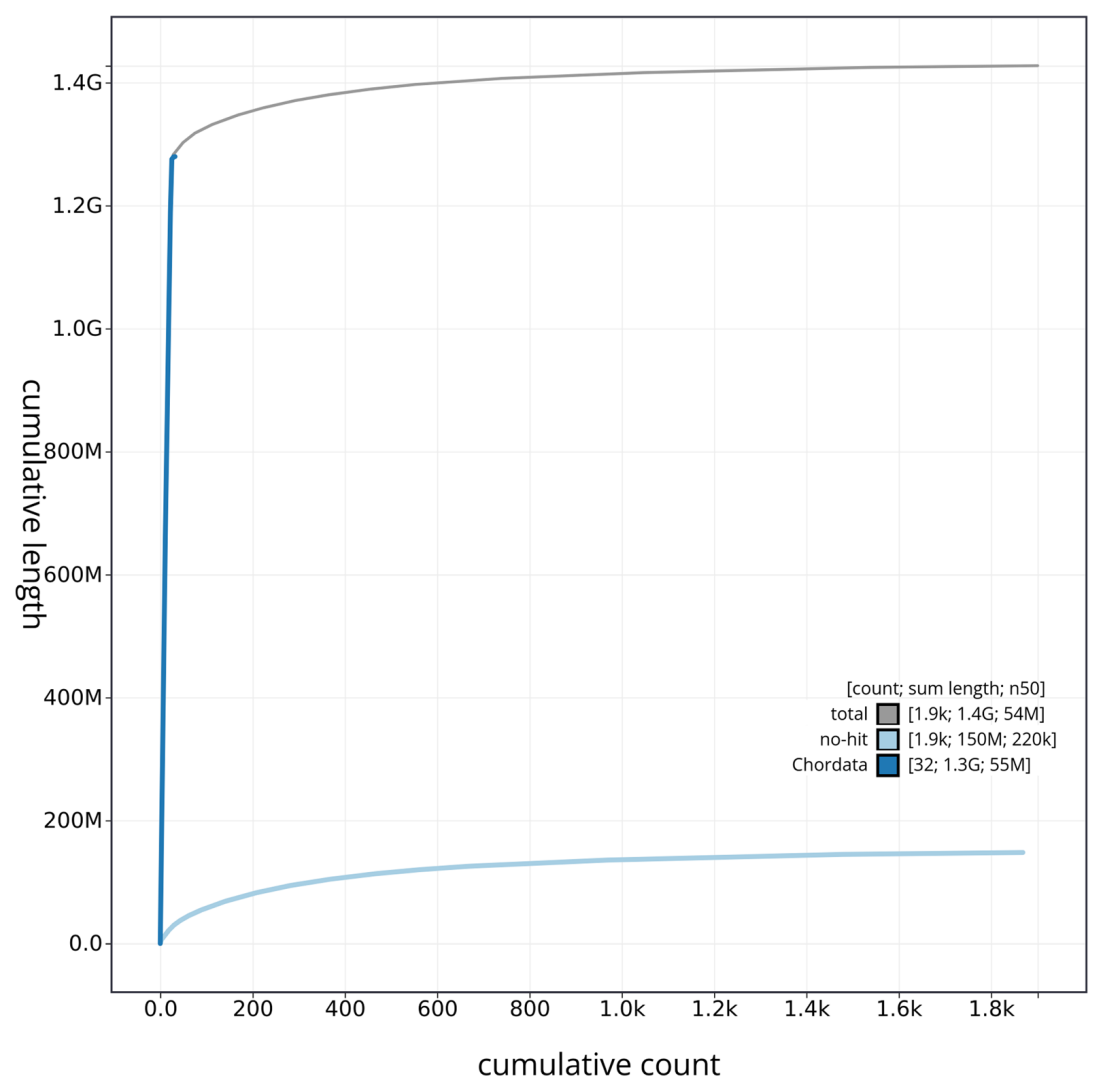
Genome assembly of *Electrona antarctica* fEleAnt2.1: BlobToolKit cumulative sequence plot. The grey line shows cumulative length for all sequences. Coloured lines show cumulative lengths of sequences assigned to each phylum using the buscogenes taxrule. An interactive version of this figure is available at https://blobtoolkit.genomehubs.org/view/fEleAnt2_1/dataset/fEleAnt2_1/cumulative.

**Figure 5.**
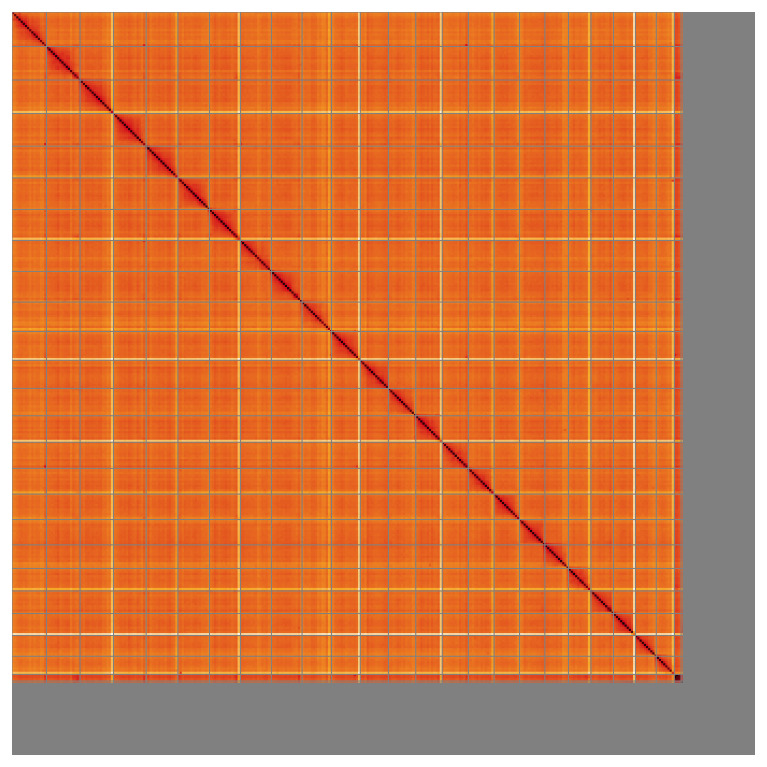
Genome assembly of *Electrona antarctica* fEleAnt2.1: Hi-C contact map of the fEleAnt2.1 assembly, visualised using HiGlass. Chromosomes are shown in order of size from left to right and top to bottom. An interactive version of this figure may be viewed at https://genome-note-higlass.tol.sanger.ac.uk/l/?d=Bmyjwb5DRka--g6D5GDvUA.

Manual assembly curation corrected 100 missing joins or mis-joins and 12 haplotypic duplications, reducing the scaffold number by 2.11%, and increasing the scaffold N50 by 2.99%. The final assembly has a total length of 1,427.40 Mb in 1,900 sequence scaffolds with a scaffold N50 of 53.6 Mb ( Table 2). The total count of gaps in the scaffolds is 3,179. The snail plot in Figure 2 provides a summary of the assembly statistics, while the distribution of assembly scaffolds on GC proportion and coverage is shown in Figure 3. The cumulative assembly plot in Figure 4 shows curves for subsets of scaffolds assigned to different phyla. Most (88.72%) of the assembly sequence was assigned to 24 chromosomal-level scaffolds. Chromosome-scale scaffolds confirmed by the Hi-C data are named in order of size ( Figure 5; Table 3). While not fully phased, the assembly deposited is of one haplotype. Contigs corresponding to the second haplotype have also been deposited. The mitochondrial genome was also assembled and can be found as a contig within the multifasta file of the genome submission.

**Table 2.** Genome assembly data for *Electrona antarctica*, fEleAnt2.1.

Genome assembly		
Assembly name	fEleAnt2.1	
Assembly accession	GCA_951216825.1	
*Accession of alternate haplotype*	*GCA_951214905.1*	
Span (Mb)	1,427.40	
Number of contigs	5,080	
Number of scaffolds	1,900	
Longest scaffold (Mb)	65.73	
Assembly metrics *		*Benchmark*
Contig N50 length (Mb)	1.0	*≥ 1 Mb*
Scaffold N50 length (Mb)	53.6	*= chromosome N50*
Consensus quality (QV)	53.3	*≥ 40*
*k*-mer completeness	Primary: 74.95%; alternate: 71.26%; combined: 96.62%	*≥ 95%*
BUSCO **	C:95.9%[S:83.1%,D:12.7%], F:1.1%,M:3.1%,n:3,640	*S > 90%, D < 5%*
Percentage of assembly mapped to chromosomes	88.72%	*≥ 90%*
Sex chromosomes	Not identified	*localised homologous pairs*
Organelles	Mitochondrial genome: 20.02 kb	*complete single alleles*

* Assembly metric benchmarks are adapted from Rhie *et al.* (2021) and the Earth BioGenome Project Report on Assembly Standards September 2024. ** BUSCO scores based on the actinopterygii_odb10 BUSCO set using version 5.3.2. C = complete [S = single copy, D = duplicated], F = fragmented, M = missing, n = number of orthologues in comparison. A full set of BUSCO scores is available at https://blobtoolkit.genomehubs.org/view/fEleAnt2_1/dataset/fEleAnt2_1/busco.

**Table 3.** Chromosomal pseudomolecules in the genome assembly of *Electrona antarctica*, fEleAnt2.

INSDC accession	Name	Length (Mb)	GC%
OX578246.1	1	65.73	43.5
OX578247.1	2	64.11	43.0
OX578248.1	3	63.62	43.5
OX578249.1	4	62.16	44.0
OX578250.1	5	60.55	43.5
OX578251.1	6	60.31	43.5
OX578252.1	7	59.04	43.5
OX578253.1	8	58.87	43.0
OX578254.1	9	58.47	43.5
OX578255.1	10	56.06	43.5
OX578256.1	11	54.82	43.0
OX578257.1	12	53.63	43.0
OX578258.1	13	52.08	43.5
OX578259.1	14	50.67	43.5
OX578260.1	15	49.8	43.5
OX578261.1	16	48.97	44.0
OX578262.1	17	48.42	43.5
OX578263.1	18	48.16	43.5
OX578264.1	19	44.95	43.0
OX578265.1	20	43.09	43.5
OX578266.1	21	42.9	43.5
OX578267.1	22	41.92	43.5
OX578268.1	23	39.46	43.5
OX578269.1	24	34.7	44.0
OX578270.1	MT	0.02	48.5

The estimated Quality Value (QV) of the final assembly is 53.3. The *k*-mer completeness was estimated at 74.95% for the primary assembly, 71.26% for the alternate haplotype and 96.62% for the combined assemblies. The primary assembly achieves a BUSCO v5.3.2 completeness of 95.9% (single = 83.1%, duplicated = 12.7%), using the actinopterygii_odb10 reference set ( *n* = 3,640).

## Methods

### Sample acquisition

Specimens of *Electrona antarctica* were collected from the Southern Ocean (latitude –53.36, longitude –38.06) on 2019-12-05. The specimen was collected by Iliana Bista and identified by Martin Collins (British Antarctic Survey) using a deep-sea net. Individual tissues were dissected from the euthanised specimen and preserved immediately in the a –80°C freezer until further processing.

The genome sequence is based on PacBio HiFi sequencing data from specimen ID SAN1100012 (ToLID fEleAnt2). A second specimen (specimen ID SAN1100013, ToLID fEleAnt3) was used for Hi-C and RNA sequencing.

### Nucleic acid extraction

The workflow for high molecular weight (HMW) DNA extraction at the Wellcome Sanger Institute (WSI) Tree of Life Core Laboratory includes a sequence of core procedures: sample preparation and homogenisation, DNA extraction, fragmentation and purification. Detailed protocols are available on protocols.io ( Denton *et al.*, 2023). The fEleAnt2 sample was weighed and dissected on dry ice ( Jay *et al.*, 2023), and tissue was cryogenically disrupted using the Covaris cryoPREP ^®^ Automated Dry Pulverizer ( Narváez-Gómez *et al.*, 2023).

HMW DNA was extracted using the Automated MagAttract v1 protocol ( Sheerin *et al.*, 2023). DNA was sheared into an average fragment size of 12–20 kb in a Megaruptor 3 system ( Todorovic *et al.*, 2023). Sheared DNA was purified by solid-phase reversible immobilisation, using AMPure PB beads to eliminate shorter fragments and concentrate the DNA ( Strickland *et al.*, 2023). The concentration of the sheared and purified DNA was assessed using a Nanodrop spectrophotometer and Qubit Fluorometer using the Qubit dsDNA High Sensitivity Assay kit. Fragment size distribution was evaluated by running the sample on the FemtoPulse system.

RNA was extracted from tissue of fEleAnt3 in the Tree of Life Laboratory at the WSI using the RNA Extraction: Automated MagMax™ *mir*Vana protocol ( do Amaral *et al.*, 2023). The RNA concentration was assessed using a Nanodrop spectrophotometer and a Qubit Fluorometer using the Qubit RNA Broad-Range Assay kit. Analysis of the integrity of the RNA was done using the Agilent RNA 6000 Pico Kit and Eukaryotic Total RNA assay.

### Hi-C sample preparation

Hi-C data were generated from the tissue from the fEleAnt3 sample, using the Arima-HiC v2 kit. In brief, frozen tissue (stored at –80 °C) was fixed, and the DNA crosslinked using a TC buffer with 22% formaldehyde concentration. After crosslinking the tissue was homogenised using the Diagnocine Power Masher-II and BioMasher-II tubes and pestles. Following the kit manufacturer's instructions, crosslinked DNA was digested using a restriction enzyme master mix. The 5’-overhangs were then filled in and labelled with biotinylated nucleotides and proximally ligated. An overnight incubation was carried out for enzymes to digest remaining proteins and for crosslinks to reverse. A clean up was performed with SPRIselect beads prior to library preparation.

### Library preparation and sequencing

***PacBio HiFi***

Libraries were prepared using the PacBio Express Template Preparation Kit v2.0 (Pacific Biosciences, California, USA) as per the manufacturer's instructions. The kit includes the reagents required for removal of single-strand overhangs, DNA damage repair, end repair/A-tailing, adapter ligation, and nuclease treatment. Library preparation also included a library purification step using AMPure PB beads (Pacific Biosciences, California, USA) and size selection step to remove templates shorter than 3 kb using AMPure PB modified SPRI. DNA concentration was quantified using the Qubit Fluorometer v2.0 (Thermo Fisher Scientific) and Qubit HS Assay Kit and the final library fragment size analysis was carried out using the Agilent Femto Pulse Automated Pulsed Field CE Instrument (Agilent Technologies).

Samples were sequenced using the Sequel IIe system (Pacific Biosciences, California, USA). The concentration of the library loaded onto the Sequel IIe was in the range 40–135 pM. The SMRT link software, a PacBio web-based end-to-end workflow manager, was used to set-up and monitor the run, as well as perform primary and secondary analysis of the data upon completion.

***Hi-C***

For Hi-C library preparation, DNA was fragmented to a size of 400 to 600 bp using a Covaris E220 sonicator. The DNA was then enriched, barcoded, and amplified using the NEBNext Ultra II DNA Library Prep Kit following manufacturers’ instructions. The Hi-C sequencing was performed using paired-end sequencing with a read length of 150 bp on an Illumina NovaSeq 6000.

***RNA***

RNA-Seq libraries were constructed using the NEB Ultra II RNA Library Prep kit, following the manufacturer’s instructions. RNA sequencing was performed on the Illumina NovaSeq 6000 instrument.

### Genome assembly, curation and evaluation

***Assembly***

HiFi reads were assembled using Hifiasm ( Cheng *et al.*, 2021) using the --primary option. Haplotypic duplications were identified and removed with purge_dups ( Guan *et al.*, 2020). Hi-C reads were further mapped with bwa-mem2 ( Vasimuddin *et al.*, 2019) to the primary contigs, which were further scaffolded using the provided Hi-C data ( Rao *et al.*, 2014) in YaHS ( Zhou *et al.*, 2023) using the --break option. Scaffolded assemblies were evaluated using Gfastats ( Formenti *et al.*, 2022), BUSCO ( Manni *et al.*, 2021) and MERQURY.FK ( Rhie *et al.*, 2020).

The mitochondrial genome was assembled using MitoHiFi ( Uliano-Silva *et al.*, 2023), which runs MitoFinder ( Allio *et al.*, 2020) and uses these annotations to select the final mitochondrial contig and to ensure the general quality of the sequence.

***Assembly curation***

The assembly was decontaminated using the Assembly Screen for Cobionts and Contaminants (ASCC) pipeline (article in preparation). Manual curation was primarily conducted using PretextView ( Harry, 2022), with additional insights provided by JBrowse2 ( Diesh *et al.*, 2023) and HiGlass ( Kerpedjiev *et al.*, 2018). Scaffolds were visually inspected and corrected as described by Howe *et al*. (2021). Any identified contamination, missed joins, and mis-joins were corrected, and duplicate sequences were tagged and removed. The entire process is documented at https://gitlab.com/wtsi-grit/rapid-curation (article in preparation).

***Evaluation of the final assembly***

The Merqury.FK tool ( Rhie *et al.*, 2020) was used to evaluate *k*-mer completeness and assembly quality for the primary and alternate haplotypes using the *k*-mer databases ( *k* = 31) that were pre-computed prior to genome assembly. The analysis outputs included assembly QV scores and completeness statistics.

A Hi-C contact map was produced for the final, public version of the assembly. The Hi-C reads were aligned using bwa-mem2 ( Vasimuddin *et al.*, 2019) and the alignment files were combined using SAMtools ( Danecek *et al.*, 2021). The Hi-C alignments were converted into a contact map using BEDTools ( Quinlan & Hall, 2010) and the Cooler tool suite ( Abdennur & Mirny, 2020). The contact map was visualised in HiGlass ( Kerpedjiev *et al.*, 2018).

The genome was analysed within the BlobToolKit environment ( Challis *et al.*, 2020) and BUSCO completeness scores ( Manni *et al.*, 2021) were calculated.

Table 4 contains a list of relevant software tool versions and sources.

**Table 4.** Software tools: versions and sources.

Software tool	Version	Source
BEDTools	2.30.0	https://github.com/arq5x/bedtools2
BLAST	2.14.0	ftp://ftp.ncbi.nlm.nih.gov/blast/executables/blast+/
BlobToolKit	4.3.7	https://github.com/blobtoolkit/blobtoolkit
BUSCO	5.4.3 and 5.5.0	https://gitlab.com/ezlab/busco
bwa-mem2	2.2.1	https://github.com/bwa-mem2/bwa-mem2
Cooler	0.8.11	https://github.com/open2c/cooler
FastK	427104ea91c78c3b8b8b49f1a7d6bbeaa869ba1c	https://github.com/thegenemyers/FASTK
Gfastats	1.3.6	https://github.com/vgl-hub/gfastats
Hifiasm	0.19.8-r587	https://github.com/chhylp123/hifiasm
HiGlass	44086069ee7d4d3f6f3f0012569789ec138f42b84a a44357826c0b6753eb28de	https://github.com/higlass/higlass
Merqury.FK	d00d98157618f4e8d1a9190026b19b471055b22e	https://github.com/thegenemyers/MERQURY.FK
MitoHiFi	3	https://github.com/marcelauliano/MitoHiFi
Nextflow	23.04.0-5857	https://github.com/nextflow-io/nextflow
PretextView	0.2.5	https://github.com/sanger-tol/PretextView
purge_dups	1.2.5	https://github.com/dfguan/purge_dups
samtools	1.16.1, 1.17, and 1.18	https://github.com/samtools/samtools
sanger-tol/ascc	-	https://github.com/sanger-tol/ascc
Singularity	3.9.0	https://github.com/sylabs/singularity
YaHS	1.2a.2	https://github.com/c-zhou/yahs

### Wellcome Sanger Institute – Legal and Governance

The materials that have contributed to this genome note have been supplied by a Tree of Life collaborator.

The Wellcome Sanger Institute employs a process whereby due diligence is carried out proportionate to the nature of the materials themselves, and the circumstances under which they have been/are to be collected and provided for use. The purpose of this is to address and mitigate any potential legal and/or ethical implications of receipt and use of the materials as part of the research project, and to ensure that in doing so we align with best practice wherever possible.

The overarching areas of consideration are:

- Ethical review of provenance and sourcing of the material
- Legality of collection, transfer and use (national and international)

Each transfer of samples is undertaken according to a Research Collaboration Agreement or Material Transfer Agreement entered into by the Tree of Life collaborator, Genome Research Limited (operating as the Wellcome Sanger Institute) and in some circumstances other Tree of Life collaborators.

## Data Availability

European Nucleotide Archive: Electrona antarctica (Antarctic lanternfish). Accession number PRJEB60649; https://identifiers.org/ena.embl/PRJEB60649. The genome sequence is released openly for reuse. The *Electrona antarctica* genome sequencing initiative is part of the Vertebrate Genomes Project (PRJNA489243). All raw sequence data and the assembly have been deposited in INSDC databases. The genome will be annotated using available RNA-Seq data and presented through the Ensembl pipeline at the European Bioinformatics Institute. Raw data and assembly accession identifiers are reported in Table 1 and Table 2.

## Author information

Members of the Wellcome Sanger Institute Tree of Life Management, Samples and Laboratory team are listed here: https://doi.org/10.5281/zenodo.12162482.

Members of Wellcome Sanger Institute Scientific Operations: Sequencing Operations are listed here: https://doi.org/10.5281/zenodo.12165051.

Members of the Wellcome Sanger Institute Tree of Life Core Informatics team are listed here: https://doi.org/10.5281/zenodo.12160324.

Members of the Tree of Life Core Informatics collective are listed here: https://doi.org/10.5281/zenodo.12205391.
