# Supplementary material for: The genome sequence of the Antarctic lanternfish, Electrona antarctica (Günther, 1878)
Source: Wellcome Open Res. 2025 Jul 25;10:89. Originally published 2025 Feb 19. [Version 2] doi: 10.12688/wellcomeopenres.23803.2 (PMC11894370; doi:10.12688/wellcomeopenres.23803.2)
Supplement: Peer review history

# Reviewer report: Reviewer response for version 2

**DOI:** 10.21956/wellcomeopenres.27236.r127706

**Published:** 2025-08-12

**Authors:** Annabel Whibley

**Affiliations:** 1 Grapevine Improvement, Bragato Research Institute, Lincoln, New Zealand

The authors have corrected the reporting error. Thank you. I have no further comments.

Are sufficient details of methods and materials provided to allow replication by others?

Yes

Is the rationale for creating the dataset(s) clearly described?

Yes

Are the datasets clearly presented in a useable and accessible format?

Yes

Are the protocols appropriate and is the work technically sound?

Yes

Reviewer Expertise:

Genomics Bioinformatics

I confirm that I have read this submission and believe that I have an appropriate level of expertise to confirm that it is of an acceptable scientific standard.

# Reviewer report: Reviewer response for version 1

**DOI:** 10.21956/wellcomeopenres.26253.r119700

**Published:** 2025-03-17

**Authors:** Annabel Whibley

**Affiliations:** 1 Grapevine Improvement, Bragato Research Institute, Lincoln, New Zealand

Bista, Collins and colleagues report the genome assembly of the Antarctic lanternfish, *Electrona antarctica*.  Relative to other DToL Genome Notes that I have reviewed, this assembly is more highly fragmented and with slightly inferior quality statistics, presumably reflecting challenges from the sample properties. It is nevertheless a high- quality assembly, with the primary assembly showing good completeness and accuracy and being robustly scaffolded into 24 pseudomolecules.

 I believe there is an error in the reporting of the total of long read sequence data that went into the initial assembly (13.66Gb data is not 43x fold coverage of a 1.4Gb genome). Reviewing the information in Table1, I think the authors have reported coverage for just one of the Sequel IIe runs, not all of them.

 The data note is well-written, with clear reporting of metadata and methods, following the DToL template. The data accessions are functional. RNA sequence data was obtained but an annotation has not yet been reported.

Are sufficient details of methods and materials provided to allow replication by others?

Yes

Is the rationale for creating the dataset(s) clearly described?

Yes

Are the datasets clearly presented in a useable and accessible format?

Yes

Are the protocols appropriate and is the work technically sound?

Yes

Reviewer Expertise:

Genomics Bioinformatics

I confirm that I have read this submission and believe that I have an appropriate level of expertise to confirm that it is of an acceptable scientific standard, however I have significant reservations, as outlined above.

# Reviewer report: Reviewer response for version 1

**DOI:** 10.21956/wellcomeopenres.26253.r119695

**Published:** 2025-03-10

**Authors:** Jayan Duminda M. Senevirathna

**Affiliations:** 1 Uva Wellassa University, Badulla, Uva Province, Sri Lanka

The study is perfect and well written in this article. The data are sufficient and nicely presented with appropriate results and publicly available.

 Hence, I would like to accept the manuscript for publication with minor modifications. Followings are suggested for verifications or improvements;

-  In Hi-C sample preparation, the frozen tissue was fixed, with what...?
- Table 2, and figure 3 titles are quiet similar.. better to change...
- "The total count of gaps in the scaffolds is 3,179." this sentence is not clear.. or maybe not necessary
-  Why you could not identify sex chromosomes, XX..?
-  In keywords; Electrona antarctica should be italic.. and keep consistency

Are sufficient details of methods and materials provided to allow replication by others?

Yes

Is the rationale for creating the dataset(s) clearly described?

Yes

Are the datasets clearly presented in a useable and accessible format?

Yes

Are the protocols appropriate and is the work technically sound?

Yes

Reviewer Expertise:

Aquatic Molecular Biology and Biotechnology

I confirm that I have read this submission and believe that I have an appropriate level of expertise to confirm that it is of an acceptable scientific standard.

# Reviewer report: Reviewer response for version 1

**DOI:** 10.21956/wellcomeopenres.26253.r119699

**Published:** 2025-03-06

**Authors:** Takushi Kishida

**Affiliations:** 1 Nihon University, Fujisawa, Japan

The authors provide a chromosomal-level phased genome assembly of an Antarctic lanternfish. This genome assembly is of high quality, and I have no more suggestions about their methodologies.

 I have only one comment about their specimens. I strongly recommend that the authors deposit the specimens used in this study (fEleAnt2 and fEleAnt3) in an appropriate museum. I think high-quality genomic data should be accompanied by vouchered specimens.

Are sufficient details of methods and materials provided to allow replication by others?

Partly

Is the rationale for creating the dataset(s) clearly described?

Yes

Are the datasets clearly presented in a useable and accessible format?

Yes

Are the protocols appropriate and is the work technically sound?

Yes

Reviewer Expertise:

Genomics, Population genetics

I confirm that I have read this submission and believe that I have an appropriate level of expertise to confirm that it is of an acceptable scientific standard.
